# Efficacy of Tamsulosin, Oxybutynin, and their combination in the control of double-j stent-related lower urinary tract symptoms

**DOI:** 10.1590/S1677-5538.IBJU.2015.0186

**Published:** 2016

**Authors:** Miguel Maldonado-Avila, Leopoldo Garduño-Arteaga, Rene Jungfermann-Guzman, Hugo A. Manzanilla-Garcia, Emmanuel Rosas-Nava, Nestor Procuna-Hernandez, Alejandro Vela-Mollinedo, Luis Almazan-Treviño, Jose Guzman-Esquivel

**Affiliations:** 1 Departamento de Urologia, Hospital Geral de Mexico, Ciudad de Mexico,; 2 Instituto Mexicano del Seguro Social Colima, no México

**Keywords:** tamsulosin [Supplementary Concept], oxybutynin [Supplementary Concept], Lower Urinary Tract Symptoms

## Abstract

**Introduction and objective:**

Indwelling double J ureteral stents are used routinely in the resolution of ureteral obstruction caused by different etiologies. Evaluation of urinary symptoms related to double-J stent, indicate that these affect 73-90% of patients. We conducted a prospective, randomized study, to evaluate the efficacy of tamsulosin, oxybutinin and combination therapy in improving the urinary symptoms.

**Methods:**

Patients who underwent ureteral stent placement after ureterolithotripsy (total 51), were randomized into three groups: Group I: Tamsulosin 0.4 mg. once per day(17 patients), Group II: Oxybutinin 5 mg. once per day (17 patients), Group III: Tamsulosin+ oxybutynin once per day (17 patients). All the groups received the drugs for three weeks and completed a Spanish validated Ureteral Stent Symptom Questionnaire (USSQ) at day 7 and 21.

**Results:**

Repeated measures ANOVA showed mean urinary symptom index score was 22.3 vs. 15.5 in group three (p<0.001) at day 7 and 21 respectively. The mean work performance index was 6.6 vs 8.1 (p=0.049) favoring tamsulosin group, the mean sexual score was 0.5 vs 1.5 (p=0.03). Among additional problems the mean was 7.2 vs 6.2 (p=0.03). No significant difference was noted among pain and general health index. No side effects were reported.

**Conclusions:**

Combination therapy with tamsulosin and oxybutynin improved irritative symptoms and work performance as well as sexual matters. Combination therapy should be considered for patients who complained of stent related symptoms.

## INTRODUCTION

The placement of ureteral double-J stents has become routine clinical practice for resolving ureteral obstruction caused by different etiologies (1-4). The estimated incidence of stent-related symptoms varies from 19-76% and includes frequency, urgency, dysuria, incomplete voiding, flank pain, suprapubic pain, urinary incontinence, and hematuria (5-10).

The objective evaluation of stent-related symptoms through the visual analog scale (VAS) and the International Prostate Symptom Score (IPSS) is complex and nonspecific (11-13). Joshi et al. developed the ureteral stent symptoms questionnaire (USSQ), which is a validated and safe psychometric instrument for evaluating the impact of ureteral stents on symptoms and quality of life. This questionnaire explores 6 areas that include urinary symptoms, body pain, general health status, work performance, sexual matters, and other additional problems (14). It has been utilized in numerous clinical trials and translated into different languages, including Spanish (15).

The efficacy of pharmacologic management of these symptoms related to the double-J stent, with alpha-1 adrenergic blockers, tamsulosin and alfuzosin (16, 12, 17-19) and the antimuscarinic agents, oxybutynin and tolterodine, has been demonstrated (20). In fact, there is a meta-analysis that evaluates the efficacy of alpha-blockers and concludes that they are associated with improvement in ureteral stent symptoms and supports their use in routine clinical practice (21). Nevertheless, at present there is no study directly comparing tamsulosin and oxybutynin. Therefore, the aim of this study was to evaluate the efficacy and safety of these two drugs for the control of lower urinary tract symptoms and their impact on quality of life.

## MATERIALS AND METHODS

### Patient selection

From November 2012 to October 2013, patients of both sexes were included in the study; they were above 18 years of age and had unilateral double-J stent placement after ureteroscopy that was performed at the Hospital General de México.

Inclusion criteria were patients of either sex above the age of 18 years that had undergone ureteroscopy for lithiasis in the lower third of the ureteral tract, with stones under 15mm, and that required unilateral double-J stent placement.

The exclusion criteria were patients with a previous diagnosis of benign prostatic hyperplasia (IPSS≥7), a previous diagnosis of overactive bladder, a history of interstitial cystitis or chronic cystitis, a history of chronic prostatitis or chronic pelvic pain, chronic medication with alpha blockers, anticholinergic agents and analgesics, ureteral obstruction caused by malignancy, pregnant patients, patients unable to understand or sign an informed consent, patients with a history of postural hypotension (decrease in blood pressure>20mmHg of the systolic or diastolic measurements) or syncope, patients with severe or unstable heart failure, severe renal failure, severe liver failure, or patients with a history of urinary retention, gastric retention, or uncontrolled wide-angle glaucoma.

A single-blind, randomized, prospective, comparative, and experimental clinical trial was conducted that included 51 patients (26 women, 25 men).

They were assigned to one of the following three groups by means of a randomization Table:

Group 1 - Tamsulosin 0.4mg PO before food every 24h for 21 days

Group 2 - Extended release Oxybutynin 5mg PO every 24h for 21 days

Group 3 - Tamsulosin 0.4mg PO before food every 24h for 21 days+oxybutynin 5mg PO every 24h for 21 days.

The sample size was calculated with the formula for comparing independent means, based on previous studies on the efficacy of alpha blockers versus placebo (20). A power of 90% and a significance level of 0.05% were used in accordance with a finite population of 60 cases (the number of ureteroscopies per year at our hospital).

### Methods

Once the diagnosis of ureteral lithiasis was established and it was confirmed that the patients met the protocol selection criteria, the characteristics of the study were explained to them. Upon accepting to participate in the study, the patients signed statements of informed consent, following the principles of the Declaration of Helsinki.

This study was approved by the institutional ethics and research committees in accordance with good clinical practices, with registration number DI/12/105/04/081.

Data were collected in a case report file that contained each patient’s personal information, demographic data, clinical history, and complete physical examination results.

Each patient underwent ureterolithotripsy and a polyurethane 24 or 22cm X 6Fr double-J stent (Cook, USA) was placed after endoscopic extraction of the stone. Adequate ureteral double-J stent placement was verified through a plain abdominal film in the immediate postoperative period.

The ureteral stent symptoms questionnaire (USSQ) was applied to all patients on postoperative days 7 and 21, registering the scores of each of the 6 topics evaluated with the assessment tool. Each section has a total score; the higher the number, the worse the general health status of the patient.

Antibiotic was administered for 7 days (Ciprofloxacin 500mg PO bid), followed by urinary antiseptic (Nitrofurantoin 100mg PO every 24h) until removal of the stent, three weeks later. Ketorolac PO 10mg was administered as needed by the patient, with a maximum of 4 tablets in 24 hours. Each patient kept a personalized register of the daily analgesic intake.

### Statistical analysis

Values were expressed as means±standard deviation (SD) and the statistical analysis was carried out using the repeated measures ANOVA test for comparing the independent means of the 3 treatment groups.

Results were considered statistically significant with a value set at p<0.05. The IBM SPSS Statistics 20.0 for Windows (SPSS, Chicago, IL, USA) statistical package was used.

## RESULTS

A total of 56 patients were enrolled in the study. Five were eliminated; four due to spontaneous stone expulsion and one for having an IPSS score above 21 points.

The remaining 51 patients (26 women and 25 men) were randomly distributed as follows:

Group 1 - Tamsulosin (17 patients)

Group 2 - Extended release oxybutynin (17 patients)

Group 3 - Combination therapy (17 patients)

Table-1 shows the demographic characteristics of the study population. There were no statistically significant differences per group with respect to age, sex, weight, height, BMI, or stone size (p>0.05).

**Table 1:** Demographic characteristics of the study patients.

Variable	Tamsulosin	Oxybutynin	Both	p*
Patients (n)	17	17	17	
Gender (M:F)	9:8	7:10	9:8	0.73
Age (years)	42.5±7.3	40.4±11.2	45.7±10.3	0.293
Weight (kg)	73.7±13.4	75±8.9	71.2±12.1	0.623
Height (cm)	166.9±5.7	161.4±5.0	161.2±5.7	0.342
BMI (Kg/m)	27.5±6.5	28.6±3.2	27.6±4.1	0.729
Stone size (mm)	8.8±3.2	11.2±4.0	10±3.02	0.135

*significance<0.05

**Figure 1:**
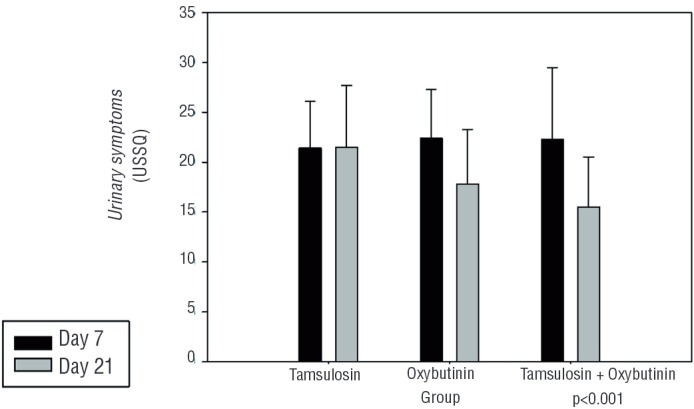
Mean urinary symptoms score.

Repeated measures ANOVA showed a mean urinary symptom score of 22.3 versus 15.5 in Group 3 (p<0.001) at days 7 and 21, respectively (Figure-1). The mean work performance score was 6.6 versus 8.1 (p=0.049), favoring the tamsulosin group, and the mean sexual performance score was 0.5 versus 1.5 (p=0.03). For the additional problems, the mean was 7.2 versus 6.2 (p=0.03). No significant difference was noted between pain and general health scores (Table-2). No side effects were reported and there were no differences in analgesic consumption between groups.

**Table 2:** USSQ score comparison among the 3 groups on postoperative days 7 and 21.

Variable		Tamsulosin (Mean±SD)	Oxybutynin (Mean±SD)	Tamsulosin + Oxybutynin (Mean±SD)	p*
**Urinary symptom score**	Day 7	21.4±4.78	22.4±4.9	22.3±7.2	<0.001*
	Day 21	21.5±6.27	17.8±5.5	15.5±5.0	
**Pain score**	Day 7	13.4±2.2	11.2±2.7	13.8±5.0	0.207
	Day 21	14.2±4.3	10.9±3.1	11.2±5.8	
**General health score**	Day 7	11.7±1.4	11.0±1.4	11.5±1.5	0.699
	Day 21	11.6±1.2	11.7±1.3	11.2±2.5	
**Work performance score**	Day 7	6.6±4.0	6.3±2.5	7.0±2.3	0.049*
	Day 21	8.1±1.8	7.2±1.9	7.7±1.5	
**Sexual performance score**	Day 7	0.6±1.1	0.5±0.8	2.6±3.8	0.036*
	Day 21	1.2±1.2	1.5±1.2	2.6±2.5	
**Additional problem score**	Day 7	8.05±2.6	6.8±1.9	7.2±1.7	0.03*
	Day 21	7.4±3.0	6.4±1.1	6.2±2.8	

*Repeated measures ANOVA.

## DISCUSSION

Ureteral stent placement for upper urinary tract diversion has been employed for more than four decades (22) and has become routine procedure in various urologic surgeries performed for different indications. Ureteral stents prevent urinary flow obstruction caused by edema of the mucosa; they aid in the mucosal healing process after a complicated procedure and they passively dilate the ureter, and facilitate the passage of residual stones (1).

Nevertheless, up to 76% of the patients will have symptoms associated with the presence of the stent, on occasion requiring its early removal (8). New stent designs, coatings, and biomaterials have been developed for the purpose of reducing these problems, but the ideal stent has yet to be produced (23).

Even though the exact pathophysiology of the symptoms related to the double-J stent is not known, it has been suggested that the mechanism involved could be an increase in the pressure transmitted toward the renal pelvis during micturition and bladder irritation due to the intravesical portion of the stent (24).

Both anticholinergic drugs and alpha-adrenergic blocking agents have been used to improve symptoms related to the double-J stent with good results in the majority of cases (20 21).

Our study showed that tamsulosin and oxybutynin combination therapy improved irritative urinary symptoms, as well as work performance and sexual aspects.

The combination of tamsulosin and oxybutynin was clearly superior in improving urinary symptoms. This is a very relevant clinical situation, given that symptomatology is present in 73 to 90% of patients with double-J stent. The same was true for the area of additional problems.

In our study, the group with tamsulosin, alone, had a higher work performance score, compared with the other two groups.

Even though there were statistically significant differences in relation to the sexual sphere, we felt that the USSQ was not very useful, especially during the first postoperative days, because the patients were more concerned about the result of the surgery and their general health than their sexual performance. In fact, the majority of patients stated that they had made no attempt at having sexual activity.

There are few controlled clinical trials that evaluate the pharmacologic agents used in the treatment of double-J stent-related symptoms, and to the best of our knowledge this is the first head-to-head study comparing long-acting tamsulosin and oxybutynin. It is well known that stent-related symptoms are similar to those of benign prostatic hyperplasia caused by urethral resistance and bladder instability. Damiano et al. reported that tamsulosin administration improved urinary symptoms and pain when evaluated through the visual analog scale, as well as quality of life (13).

Wang et al. stated that the selective alpha-1 blocker, tamsulosin, improved urinary symptoms, flank pain, and pain during micturition (19). Beddingfield et al. reported that patients treated with 10mg daily of alfuzosin showed improvement with respect to micturition frequency, lumbar pain, and sleep disorders (25). Deliveliotis et al. had similar study results of improved stent-related symptoms in patients treated with alfuzosin, especially in reference to pain, as well as sexual function, and general health (26).

Symptoms associated with double-J ureteral stent are similar to those of overactive bladder caused by involuntary bladder contractions, and antimuscarinic agents have been used with good results (27). Norris et al. reported that there were no statistically significant differences between patients treated with oxybutynin compared with placebo or phenazopyridine (28). Agarwal et al. demonstrated that patients that had received oxybutynin or tolterodine prior to surgery showed greater relief in regard to bladder discomfort compared with patients in the placebo group (29).

A limitation of our study resulted from the fact that even though the sample size was adequately calculated, the number of patients in each group was small and this could possibly reduce the capacity to detect other potential effects of these drugs in patients with double-J stent, when evaluated through the USSQ.

## CONCLUSIONS

The administration of tamsulosin and oxybutynin markedly reduced the irritative symptoms commonly associated with a double-J stent. These drugs were also effective in the improvement of sexual performance and work performance, as well as other related problems. There was no difference with respect to pain and general health.
